# Mapping meanings: methodological innovations to restore memories of place in a public park in Santiago de Chile

**DOI:** 10.3389/fpsyg.2025.1726748

**Published:** 2026-03-09

**Authors:** Teresa Ropert, Ana Rosenbluth, Martín Nilo, Luis Valenzuela, Michelle Bernardino

**Affiliations:** 1 School of Psychology, Escuela de Psicología, Universidad Adolfo Ibáñez, Santiago, Chile; 2 Escuela de Gobierno, Universidad Adolfo Ibáñez, Santiago, Chile; 3 Independent Researcher, Río de Janeiro, Brazil

**Keywords:** critical cartography, environmental psychology, geo-indexicality, place-assemblage, qualitative methods, visual methods

## Abstract

In its conventional conception, the map has been defined as a “map-instrument,” that is, as a technical device of an informative-practical nature. However, from the perspective of critical cartography, representation is understood rather as a spatial discourse, capable of “producing” political images of the territory and integrating subjectivity as one of its constitutive elements. In parallel, environmental psychology has long examined how people construct places through their interactions with and modifications of their environment, thereby linking places to meanings and to personal and collective experiences. Traditional approaches within this field have increasingly been questioned for their apolitical and uncritical assumptions, underscoring the need to recognize that the person-place relationships emerge from the interaction of personal, social, and political-contextual dimensions. This critique resonates with challenges posed by critical cartography to positivist approaches to map production, emphasizing how individuals and groups actively construct their social and spatial reality. This paper builds on data produced as part of a study conducted in Quebrada de Macul Park in Santiago de Chile, with the aim of developing a novel methodological approach to the study of place-assemblages. Place-assemblages are understood here as an ontological concept for examining analytical entities that intertwine material, symbolic, and practical dimensions of people-place relationships. Our guiding research question is: (How) Can subjective and/or psychosocial processes embedded in people-place relations be cartographically represented? To address this question we conducted (i) a systematic literature review to synthesize recent publications that integrate psychosocial and/or narrative processes with cartographic representations, and (ii) a reflexive thematic analysis of two in-depth interviews from the original study aimed at understanding how narratives might produce diverse multi-layer cartographies through which place is conceived as socially and politically intertwined. In doing so, the paper aims to advance methodological strategies, as well as onto-epistemological discussions, on how to operationalize the ontology of ‘place-assemblages’, translating this conceptual proposal into a concrete framework for capturing the symbolic, material, and practical nature of the person-place’s unity.

## 1 Introduction

Beyond their apparent neutrality, maps have historically functioned as political artifacts: they served nationalistic meta-narratives, delineated strategic spaces, legitimized state control, and facilitated the inclusion or exclusion of populations (Caquard, 2013; Peluso, 1995; Santos, 1977a; Santos, 1977b). Critical geography and critical cartography have challenged this positivist legacy by exposing the social imaginaries and power relationships embedded in cartographic practices (Harley, 1989; Pickles, 2004). From this perspective, maps are not merely technical representations but components of broader spatial discourses through which institutions define, classify, and regulate territory. This position resonates with theoretical traditions that conceptualize space as socially produced and politically mediated. Santos (1977a) and Santos (1977b), for example, argues that space is shaped through unequal social and economic relations, while Lefebvre (1991) contends that representations of space—such as maps—are central instruments in the production of abstract, homogeneous spatial orders aligned with the interests of the state and capital. From this perspective, cartographic practices do not merely depict territory; they actively participate in shaping territorial imaginaries and modes of spatial governance, privileging particular ways of seeing and organizing space over others. Parallel to these debates, environmental psychology has long examined people–place relations, highlighting how places are actively constructed through interactions that generate meanings, attachments, and identities (Proshansky, 1978; Vidal and Pol, 2005; Lewicka, 2011). While traditional approaches emphasized affective and cognitive bonds with places, more recent work has revealed their ambivalence, especially in contexts of inequality and exclusion (Manzo, 2005, 2014; Ropert and Di Masso, 2020).

With regard to cartographic approaches, cognitive mapping stands out as an emblematic tradition, particularly significant in the consolidation of environmental psychology (Pol, 2006), at a time when representational conceptions of the mind were still dominant within mainstream psychology. In the early 20th century as environmental psychology began to emerge, the call for an ecological approach to human behavior paradoxically led to a strong focus on cognitive dimensions of people-place relationships, even before the field was formally recognized as a distinct subdiscipline (Pol, 2006).

Today, although environmental psychology encompasses a broad range of research methods, the literature continues to be dominated by action-oriented models, quantitative survey studies, and correlational analyses of behavioral determinants (Kuhn and Bobeth, 2022), with publication largely concentrated in Anglo-American contexts (Xu et al., 2018). In response, growing strands of research have called for the adoption of critical epistemologies and alternative methodological approaches – often qualitative in nature- to better account for how political, historical, and power relations, as well as social injustices, profoundly shape people –place relationships. These perspectives emphasize that environmental psychology can no longer be understood from an exclusively individualistic perspective (Di Masso et al., 2008; Kuhn and Bobeth, 2022; Manzo, 2005, 2014; de Pinto Carvalho et al., 2025).

Contested spaces is a core idea of Henri Lefebvre’s seminally theorization of the social production of space: meaning that space is generated through as the outcome of intertwined relation between everyday spatial practices, expert representations of space and lived experienced meanings, rather than just a pre-given support for social life (Lefebvre, 1991). While Lefebvre emphasizes an European production of space through perceived, conceived and lived dimensions, Brazilian geographer Milton Santos, develops a complementary Latin-American perspective, conceptualizing space as a relational totality constituted by the interplay of form, function, structure and process, insisting on the historical–material articulation between techniques, everyday life and global capitalism (Santos, 1977a; Santos, 1977b; Santos, 1997; Santos, 2000).

Both fields converge on a central insight: places are not neutral containers but contested, lived, unequal, and socially mediated spaces. Despite their shared understanding, critical cartography and environmental psychology have rarely been brought into direct methodological dialog. This article seeks to address that gap: research with emphasis on space as a concrete configuration of forms, functions, structures and processes informs our attention to how cartographic representations can render visible the socio-technical and political-economic dimensions of these place-assemblages. We bring (emergent critical) environmental psychology into conversation with critical cartography in order to explore how subjectivities and psychosocial processes can be represented cartographically. The focus is on place as a psycho-socio-political and discursive construct, and on critical and participatory cartographies as methodological tools for visualizing and interrogating person-place relationships.

### 1.1 Place as a psycho-socio-political and discursive concept

Across disciplines, the concept of place has developed along distinct yet intersecting trajectories. Environmental psychology and human geography offer complementary but differently inflected accounts (Tuan, 1977; Relph, 1976; Lefebvre, 1991; Agnew, 2011; Proshansky, 1978; Proshansky et al., 1983).

First, environmental psychology has traditionally examined the affective, cognitive, and identity dimensions of people–place bonds. Seminal work on place attachment (Altman and Low, 1992; Giuliani and Feldman, 1993; Scannell and Gifford, 2010; Lewicka, 2011) and place identity (Proshansky, 1978; Proshansky et al., 1983; Twigger-Ross and Uzzell, 1996) conceptualizes these ties as multidimensional relationships involving emotions, cognitions, and behaviors. Later studies elaborated on these constructs (Bernardo et al., 2023; Droseltis and Vignoles, 2010; Lewicka, 2011; Scannell and Gifford, 2010; Hidalgo and Hernández, 2001) concluding that (i) place attachment means that people need to stay close to meaningful places, whereas time of residence, geographical scale, community ties, and its physical characteristics, among others, are crucial determinant factors; and (ii) place identity might contribute to satisfy psychological needs and drives, like self-esteem, self-continuity, self-efficacy, distinctiveness and belonging, among others. In all this work, one big conclusion is that people become aware of their attachment and identification with places when they are somehow jeopardized, by mobility, natural disasters, and/or urban changes. This line of research demonstrates that places are socially constructed through interaction, environmental modification, and the attribution of meanings at individual and collective levels (Proshansky, 1978; Vidal and Pol, 2005).

More recently, critical strands within environmental psychology have emphasized the socio-political character of people–place relations. Rather than treating attachment and identity as cognitive or mainly individual constructs, these approaches situate place within dynamics of inequality, exclusion, and contestation (Di Masso et al., 2008; Manzo, 2005, 2014). Place is understood as embodied, affective, and socioecological, following Di Masso and Dixon’s (2015) critique of the internal/external divide (de Pinto Carvalho et al., 2025). Thereby, place is constituted through discourses, practices, and materialities, entangled with power relations, social norms, exclusion dynamics, subjectivity, and collective actions, needing new epistemological and methodological approaches to its study (e.g., de Pinto Carvalho et al., 2025; Ropert et al., 2022).

Such perspectives challenge romanticized notions of place and highlight how place-based bonds are entangled with broader struggles over recognition, belonging, and the right to space (Di Masso et al., 2014; Manzo and de Pinto Carvalho, 2020; Ropert and Di Masso, 2020). In this sense, environmental psychology can directly converse with critical geographical debates, as both emphasize that place is discursively and materially constructed, contested, and represented. From a critical-discursive approach, this implies that place identities are not fixed traits, but psycho-political processes embedded in everyday talk and practice (Di Masso, 2012; Di Masso and Dixon, 2015; Dixon and Durrheim, 2004). Recent work on resistance to desegregation extends this insight by showing how socio-spatial boundaries are defended through constructions of “boundary transgression” and perceived ruptures of the spatial order, illustrating how place identities are bound up with practices of segregation, avoidance, and the policing of who is “out of place” (Dixon et al., 2025).

Similarly, foundational contributions of human geography by Tuan (1977), Relph (1976), and Massey (1994, 2005) frame place as lived experience, as a counterpoint to homogenizing forces, and as a node in relational, power-laden networks. Lefebvre’s (1991) theory of the production of space and Agnew’s (2011) account of place and politics further underscore that space/place is never neutral but historically and ideologically constituted. Within Lefebvre’s framework, the notion of “counter-space” designates spatial practices and representations that emerge in opposition to abstract and homogenizing spaces of power, prefiguring contemporary discussions of counter-geographies and counter-cartographies. Relatedly, scholars highlight place as socially inscribed through norms and boundaries that regulate belonging and practice, which is brilliantly expressed in the popular saying of ‘to be out of place’ (Cresswell, 1996; Gieryn, 2000). Urban places, then, are both socially constructed and politically contested, delineating who has the right to use them and how. At the same time, places are dialectically anchored in subjectivity, shaped by the mutual influence between people and groups and the meanings and representations they attribute to specific spaces (Anderson, 2004).

Assemblage thinking further contributes to this picture by conceptualizing place as a dynamic, multi-scalar entanglement of material elements, social practices, and discourses. Rather than seeing place as a bounded entity, assemblage approaches emphasize flows, connections, and emergent properties, offering a way to reconcile phenomenological and social-constructionist understandings of place (Dovey, 2020). This resonates with environmental psychology’s transactional view and anticipates later conceptualizations of place-assemblages in critical person–place research (Altman and Rogoff, 1987). Hence, we understand places as constituted through affect, identity, discourse, and power, whereas meanings, exclusions, and forms of belonging are continually negotiated, considering the psycho-socio-political dynamics of people–place relations.

### 1.2 Critical and participatory cartographic approaches to person–place relations

Historically, scholars conceived cartography as a scientific and objective theory, defining the map as a “map-instrument”—a technical device of an informative and practical nature that reflected the territory in an impartial and neutral way (Crampton and Krygier, 2010; Pérez and Vicencio, 2024). The foundational contributions of Harley (1989, 1990), Wood (1992), and Pickles (2004) consolidated what is known as *critical cartography*, which understands maps not as neutral mirrors of space but as spatial discourses imbued with power. From this perspective, cartographic representation is capable of producing political images of territory and incorporating subjectivity as one of its constitutive elements (Montoya, 2007; Wood and Fels, 2008). Thus, critical cartography challenges the positivist pursuit of “objective” map-making, arguing the pursuit of objective representations and calls for repositioning “the role of social imaginaries in the production and reproduction of social life” (Pérez and Vicencio, 2024, p. 21).

Within this critical field, diverse methodological practices enact its principles in concrete settings. Counter-cartography (or *counter-geographies*), as articulated by Peluso (1995) and later Kollektiv Orangotango+ (2023), refers to mapping practices explicitly designed to defend rights and support struggles for social justice. These cartographies seek to delegitimize dominant discourses and reclaim territories for marginalized or silenced groups. Roux (2023) emphasizes that such maps should not be reduced to their visual dimension: like stories, they are intentional acts that both describe and contest reality, becoming tools of resistance against capitalism, colonialism, and extractivism.

Counter-mapping, on the other hand, is often used more narrowly to describe grassroots mapping practices developed in direct confrontation with state or corporate cartographies. Dalton and Mason-Deese (2012) and Mason-Deese (2020) conceptualize it as a form of militant research, where mapping links knowledge production to grassroots activism and collective resistance. To achieve these objectives, counter-cartography draws on tools from academic research, field practice, political activism, and art, consolidating maps that stand out for the plurality of voices, the integration of narratives, and the use of diverse visual strategies to represent subjectivity and collectivity (Kollektiv Orangotango+, 2023).

Although the antecedents of critical cartography reach back into the history of geography, it was in the 1990s—particularly with innovative exhibitions such as De Bois’ Paris show representing the condition of Black communities in the United States (Du Bois, 1900)—that a more explicit embrace of critical approaches gained traction. Current examples include participatory or social cartography, defined as the collective construction of spatial representations to mobilize communities around collective action at local scales (Chambers, 2006; Sieber, 2006; Goodchild, 2007). Increasingly, scholars also refer to narrative cartographies or “story maps,” understood as any cartographic representation that incorporates narrative elements (Roth, 2021) or as “the graphic representation of the spatial configuration of the world represented by the text” (Ryan, 2020, p. 1). Narrative, as Roth (2021) emphasizes, involves structuring stories in ways that shape both individual and collective identity. Recent “deep mapping” initiatives push narrative cartography further by integrating heterogeneous data—material remains, textual sources, oral histories, and spatial analyses—to produce “thick descriptions” of place that highlight its temporal depth and multi-vocality (Katevaini and Williamson, 2025).

In dialog with Lefebvre, for Santos, territory is never neutral and much less a solely support, but the configuration of systems of objects and actions historically produced, throughout unequal power relations and between conflicts. In this sense, counter-geographies emerge when every day, popular and marginalized practices appropriate and resignify territories against their dominant, institutional, corporate or state-oriented uses, giving rise to banal or popular spaces where alternative rationalities and values sustain a new territorial meaning. Santos approach to counter-cartographies maps the “used territory” from below, making visible the dense mesh of practices, solidarities and memories that contest the abstract, globalized spatial logic of capital; therefore, not simply oppose official geographies. Such critical cartography exercises assess registering the popular and activist uses a living territory, thereby reinforcing counter-geographical projects that challenge its reduction to a homogeneous space.

In Latin America, although the label “critical cartography” is less commonly used, practices akin to counter- and participatory mapping have long emerged as tools to challenge conventional cartographic paradigms and to articulate locally grounded territorial struggles. These practices have been mobilized to resist extractivism, state territorial control, and hegemonic representations of space, while foregrounding local knowledge and collective agency (Ospina Mesa, 2023). For example, the Colectivo de Geografía Crítica del Ecuador (2019) advances the notion of *pluriterritoriality*, demonstrating how mapping can both contest dominant territorial logics and imagine alternative socio-spatial futures.

Concrete initiatives includethe Projeto Nova Cartografia Social da Amazônia in Brazil (Almeida and Farias, 2013), the Iconoclasistas collective in Argentina, and Geocomunes in Mexico, as well as transnational compilations such as *This Is Not an Atlas* (Kollektiv Orangotango+, 2018, 2023). Together, these experiences suggest that critical cartography in the region is best understood not as a unified theoretical framework but as a plural field of participatory, collaborative, and counter practices. These practices challenge dominant cartographic authority while generating situated imaginaries of territory and place (Sletto et al., 2020; Piazzini Suárez and Montoya Arango, 2022).

Recent academic work in Latin American cities further illustrates this potential. In Quito (Ecuador), counter-cartographic approaches have been used to render visible “invisible networks” of dissident spatial practices, such as those documented in La Jota Street, foregrounding everyday forms of appropriation and resistance that remain obscured in official cartographies (Cano-Ciborro and Medina, 2023). Similarly, research on urban resignification processes in the Barranco area of Cuenca (Ecuador) shows how historical layers, commodification, and tourism-driven interventions reconfigure the meanings of central urban areas, generating spaces of dispute where past inscriptions and present appropriations collide (Jiménez-Pacheco et al., 2025).

### 1.3 Environmental psychology and cartographic approaches: visualizing person–place relations

A persistent challenge in environmental psychology has been how to move beyond verbal accounts and experiment with visual and cartographic ways of representing people–places relationship. With the discursive turn and the growing demand to politicize environmental psychology, new initiatives have foregrounded narratives, discourses, and meanings through visual representation. Photographs, combined with interview excerpts and other narrative techniques are particularly common (Di Masso and Dixon, 2015; Manzo, 2014). Maps themselves are sometimes employed to delimit and characterize spaces of analysis from a psychosocial perspective (e.g., Berroeta and Vidal, 2012; Dixon and Durrheim, 2004), and particularly interesting the recent work of Dixon et al. (2020) and Dixon et al. (2022) mapping social groups over places, and analyzing daily mobility practices tending to avoid the ‘outgroup’. All in all, even studies that explicitly seek methodological innovation—such as walking interviews—often privilege written accounts over other forms of visual communication (Anderson, 2004; Jones et al., 2008). A few exceptions stand out: Clark and Emmel (2010) integrate mapping with photography, while Evans and Jones (2011) combine cartography with narrative accounts to render place relations more dynamically.

Recent developments in environmental psychology and related fields further expand the repertoire of visual and cartographic approaches. Affective mapping techniques, such as the Instrumento Gerador de Mapas Afetivos (IGMA), invite participants to draw and annotate maps that capture affective images of their environments (Pinheiro Pacheco and Cruz Bomfim, 2025). These methods reveal how residents’ experiences of agreeableness, belonging, and contrast become spatially organized, especially in contexts of threat, expropriation, or urban transformation, and thus provide a concrete way of visualizing the psycho-affective dimensions of person–place relations. Studies of activity spaces in urban environments similarly combine mobility data and perception measures to compare “statistical” diversity with perceived diversity, highlighting how contact patterns and affective ties shape spatial experience beyond what can be inferred from census data alone (Holubowska et al., 2025). In parallel, research on historical and educational settings has used spatial analysis and narrative interpretation to explore how architectural configurations, visibility patterns, and symbolic elements foster cognitive mapping, identity, and emotional cohesion (Shi and Tian, 2025).

Building on these critiques, Di Masso and Dixon (2015), drawing on the affective–discursive lens (Wetherell, 2012) and research on the politics of public space (Durrheim et al., 2013), propose the concept of the *place-assemblage*: an “ever-shifting but temporarily stabilized entanglement of spatial arrangements (including geographical locations, physical objects and spatial boundaries), embodied practices, and discursive constructions (i.e., socially organized and oriented patterns of language use) of environment and people–place relations” (p. 49). This perspective refuses to privilege language as the only window onto place. Instead, it highlights how affect, bodily routines, infrastructures, and discourses, co-constitute people–place relations, dynamically shaping and reshaping them. It converges with assemblage-based accounts of place in human geography that emphasize relationality, multiplicity, and emergence (Dovey, 2020).

It calls on environmental psychology to move beyond words and capture the meanings of place through multimodal evidence: traces and routes (e.g., walking methods), photographs and situated narratives, observations of practice, and, crucially, spatial representations that show how bodies, artifacts, and boundaries configure everyday life (Di Masso and Dixon, 2015). From this standpoint, critical and participatory cartographies emerge as natural allies. By integrating diverse modalities and perspectives, mapping can materialize the entanglements of the place-assemblage, making visible attachments, exclusions, and territorial claims that otherwise remain hidden. Deep mapping initiatives, in particular, demonstrate how GIS, story maps, and layered data can be mobilized to reconstruct complex senses of place, capturing temporal depth, multiple voices, and the interplay between material and symbolic dimensions of space (Katevaini and Williamson, 2025). Such approaches provide a promising framework for visualizing the psycho-socio-political and discursive nature of people–place relations that this article seeks to address.

## 2 Case study and context

A narrative-inspired study conducted in Quebrada de Macul Park employed mixed methods (in-depth interviews, participant observation, as well as quantitative analysis and mapping of secondary data) to describe and interpret the meanings and uses associated with the park (Rosenbluth et al., 2024). Findings revealed that the park functioned as a profound identity marker for long-term residents, particularly a group that spearheaded a citizen movement to reclaim the park when its access was threatened by a large-scale real estate project. For these residents, the Quebrada embodied the identity of Peñalolén and thus had to be defended against closure.

As underlined, this article is taking up again research conducted in the Quebrada de Macul Natural Park, in the eastern metropolitan fringe of Santiago city, in Chile, in the slope of the Peñalolén municipality’s Andean Mountains. The park is a city link with the Andean foothills, by a network of ravines, water streams, and hiking trails. Peñalolén is a mainly residential municipality, with a population of approximately 270,000 inhabitants in 2024, which corresponds to 4–5% of the total metropolitan population. It presents a wide degree of socio-spatial heterogeneity, including high and middle-class neighborhoods, social dwellings, and traditional Popular Sectors, as well as community associations, which have a strategic role in the environmental and territorial struggles in this locality.

Long before being a park, Quebrada de Macul had been a visited spot for locals in an informal way as a natural recreational space to the Andean Mountains. However, in early 2000s, a large real estate project threatened to cut public access to Quebrada de Macul, which led to community organization in support of demands for the preservation of Quebrada de Macul as a public asset. As a consequence of this struggle and subsequent negotiations, it was proposed to establish a municipal natural park with a lease term to consolidate Quebrada de Macul as a metropolitan mountain park and symbol of community resilience. Present-day Quebrada de Macul is visited by people all over Santiago, being a main symbol of identity for inhabitants of Peñalolén in addition to being a place where memories of its everyday usage, struggles in defense of nature, and access to nature in the city have been produced.

Today, Quebrada de Macul Park receives approximately 147.390 visitors annually in average from across Santiago, other Chilean cities, and abroad (Rosenbluth et al., 2024). Yet its present status as a metropolitan public park tends to obscure the history of local residents’ struggle to secure access. Against this backdrop, we developed a critical cartographic exercise to visualize the memory processes inscribed in the history of Quebrada de Macul. The next section details the methodology of this exercise, as well as its limitations and directions for future research.

Moreover, since its declaration as a free public park under the administration of the Municipality of Peñalolén, however, processes of massification have produced a sense of *place nostalgia* or *solastalgia* (Albrecht, 2006; Albrecht et al., 2007), a form of distress generated by transformations to a place felt as one’s own.

## 3 Methodology

As pointed out, this paper revisits research conducted between October, 2023 and January 2024 in de district of Peñalolén, in Santiago de Chile. The original project involved six in-depth interviews and five participant observation sessions aiming at understanding the varied uses and meanings, social and ecological activists, longtime residents, and park employees attribute to the Park Quebrada de Macul, In the present paper, we revisit part of these data to develop a critical mapping methodological exercise aimed at understanding the memory processes that have shaped the history of the Quebrada de Macul, and transformed it through the actions of politically active neighboors who reclaimed the place as their own. In doing so we adopt ‘place-assemblages’ as the ontological foundation of our methodological proposal.

### 3.1 Systematic review methodology

We conducted a systematic literature review following De León-Casillas et al. (2020)‘s guidelines for Social Sciences. Unlike classical reviews focused on medical or health intervention outcomes, our aim was to identify and systematize methodological advances in narrative cartography for representing people’s subjectivity and psychosocial processes. To this end, we formulated the following guiding research question: Which recent publications on psychosocial and/or narrative processes of specific groups linked to a territory include cartographic representation as part of their results?

Inclusion criteria were as follows: (a) papers in written in English, Spanish or Portuguese (corresponding to the linguistic competencies of the Chilean-Brazilian research team); (b) studies that included cartographies or maps as part of their results; and (c) empirical research. Initially, we restricted the review to the last 5 years, but this criterion was removed after observing an insufficient number of eligible publications.

Searches were conducted in three academic databases: Scopus (*n* = 92), Web of Science (WoS) (*n* = 37), and SciELO (*n* = 17). We used the following keyword combinations in English, Spanish, and Portuguese: *“cartography AND psychosocial”* and *“cartography AND qualitative analysis.”* These terms were refined through multiple team discussions to ensure conceptual accuracy.

The selection process consisted of five stages, all conducted by two independent researcher. Any disagreement or uncertainty was discussed and resolved collectively by the full research team:

1. Concept refinement: The research team held multiple meetings to define the key search concepts and finalize keyword combinations.
2. Initial search: Articles were retrieved from Scopus, WoS, and SciELO using the defined search terms.
3. Rapid screening: We excluded articles that were non-empirical or did not include cartographies or maps in their results (*n* = 44).
4. Duplicate removal: Duplicated records were removed (*n* = 39).
5. Full-text review: We selected articles that worked with assembled material-symbolic research objects, where spatial representations condensed the voices of participants or groups (*n* = 8).

**Figure 1:**
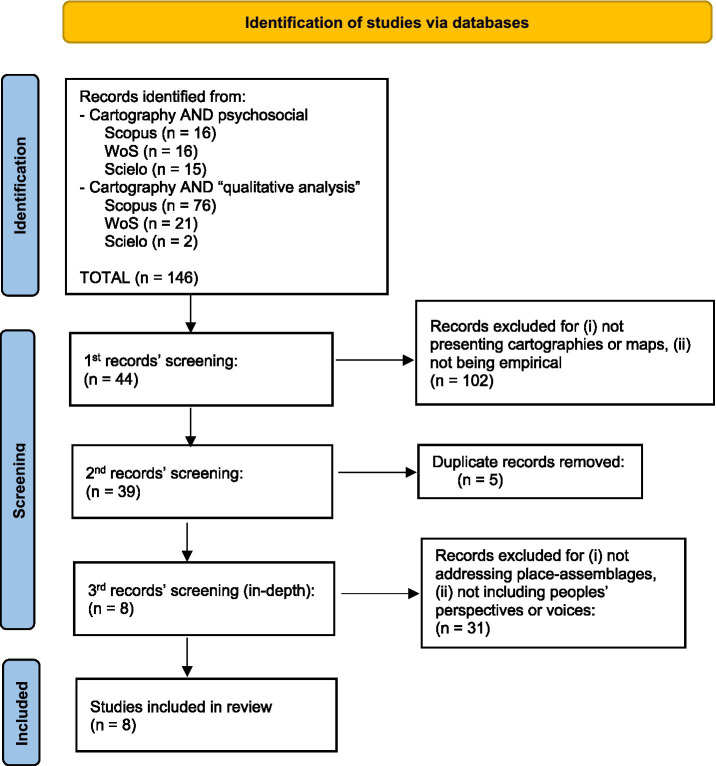
Flow diagram for systematic review’s results based on Prisma (2021).

A flow diagram based on Page et al. (2021) (Figure 1) summarizes this selection process, and Appendix A provides the selection table to ensure reproducibility. We organized the findings in three analytical tables focusing on methodological approaches to producing subjective maps: (i) Table 1 general characteristics of each study (authors, title, year, journal scope, and country of data collection); (ii) Table 2 methodological information (epistemology, design, sign, sample size and characteristics, data collection, methods, analytical strategies, quality assurance, and the use of technological vs. non-technological tools for achieving place-assemblages, e.g., geo-indexation or hand-drawn representations); (iii) Table 3 main results and discussions, including a critical analysis of the interpretive themes identified in each article.

**Table 1:** Sociodemographic information of participants.

Pseudonym	Age	Gender	Profile	Interview n°	Duration	N° Unit of Analysis
Bernardita	79	Woman	Community leader and activist	1	1:44:15	42
Hernán	32	Man	Longtime resident and tour guide	4	1:11:47	101

**Table 2:** Georeferencing of ‘place-assemblages’s points for final map construction.

Referenced place	LAT	LON	Qualitative category	Emotionality	Narrative (English)
Reservoir	−33,49,308	−70,5,173,787	Historic hill	Positive	“It was beautiful, it was countryside, fields and hills. And the ravine. And we went to the ravine because there was a reservoir where the parking lot is now” (B).
Small field	−33,4,827,383	−70,5,204,608	Actual hill	Positive	“There was a place called El Campito (small field), which was nice because it was below the ravine […] and in September it would fill up with kites. It was beautiful, gorgeous. Even now, people still sneak into the university” (B).
Sapolio Cave	−33,4,855,273	−70,5,203,224	Historic hill	Nostalgic	“It was like a cave. There were lots of myths about it, from what I heard: “No, it’s a caveman’s cave,” “There was sapolio in the cave.” […] We passed by his university and it’s there, but it’s covered up, […] they covered it up because they were going to party. […] It still had its mystique because climbing up there was a huge effort” (H).
Cows and horses	−33,487,389	−70,5,171,119	Historic hill	Nostalgic	“There is a nearby, there is a guard, and there are other university buildings. But there were many animals there, cows, horses, everything” (H).
Tree burning	−33,4,999,324	−70,5,097,948	Historic hill	Enraged	“Those who burned his horses caused such great damage, 40 horses were burned, they burned forests that were there and were not there, and worst of all, they are native trees… people out of malice, out of malice” (B).
Logged forest	−33,5,013,851	−70,5,099,765	Historic hill	Nostalgic	“There used to be a forest there, it was very beautiful, and then they cut it all down. Well, the people who went there sometimes did a lot of damage too, but there were reasons for that… They started fires, where they made campfires to cook” (B).
Pampa	−33,4,902,224	−70,5,176,988	Historic hill	Neutral	“You climbed up the hill a little and there was a meadow where there was already water, where there were already animals” (H).
Alternative paths	−33,5,102,129	−70,4,834,414	Actual hill	Neutral	“And there’s also one on the left, and you can go that way too—that’s how it was done in the old days because nobody knew how to get through there—it’s shorter but more dangerous because if you fall, you’ll kill yourself” (H).
Park entrance	−33,4,924,053	−70,5,182,972	Actual hill	Neutral	“Poldi got himself a tent, I do not know, they gave him a military tent, one of those army tents, and we held our meetings in the tent, of course, with the owners from over there who claimed to be the owners of the ravine” (B).
Iron gate	−33,5,005,344	−70,5,113,435	Actual hill	Positive	“When we inaugurated the project that won us all that, they put up a huge, beautiful iron gate. Poldi thought it would be a good idea to inaugurate it and invite the authorities to come” (B).
Lion King Stone	−33,5,270,966	−70,4,904,865	Actual hill	Positive	“If you take the path to the right, we call it the path of the lion king rock, which is a rock that looks like this, with another rock on top and a cave underneath. So you climb up, you can stand in the cave or stand on top, and it has a point, and you can see the whole ravine” (H).
River pools	−33,5,008,492	−70,5,088,137	Historic hill	Nostalgic	“The pools in Quebrada Macul from before, from long ago, are nothing like they are now. Here, you get in and the water comes up to your waist, if you are lucky. But in those days, people would put rocks in make pools. […]” (H) “It took about 2 h to put big rocks and everything in place to make the pool so we could bathe […]. Not now, though. Now there’s so little water that it’s sad.” (B)
Second waterfall	−33,4,986,715	−70,4,819,446	Actual hill	Positive	“You see people who are really very spiritual. Suddenly they arrive at this waterfall and there are people who have their… I’ve seen Buddhists, I’ve seen religious people, Catholics who sit by the waterfall and start reading their Bible. So, in itself, just as the waterfall is like a more sacred place…” (H).
First waterfall	−33,5,002,849	−70,4,845,107	Actual hill	Positive	“From 2005 to around 2010, they were fixing up the Quebrada, putting in railings and repairing the path, because as you climbed up to the San Juan waterfall, it wasn’t as easy as it is now. […] There were paths that were very narrow, with precipices below. So they widened them, placing stones” (H).
University Adolfo Ibáñez	−33,4,909,645	−70,5,128,028	Actual hill	Nostalgic	“When they started building the university, for example, the cave disappeared. It no longer exists. And they began to close off all the paths that were not marked, so now you can only follow one route to get there” (H).

**Table 3:** Identification and characterization of selected papers.

ID paper	Title	Authors	Year	Journal	Journal scope (webpage)	Country of reference
1. Mapping migration	Mapping migration detention: Mixed methods, grounded theory, transdisciplinary encounters	Juliaa Manek, Amyb Nethery, Francescac Esposito, Paue Pérez-Sales, Horz Holgera	2023	Methods in Psychology	Psychology	Mexico, Sano
2. Weaving nests	Weaving nests, daring to fly: production of multiplicities in the territories of users of Psychosocial Care Centers	Nathália Ferreira de Souza e Silva	2022	Ciência & Saúde Coletiva	Public Health	Brazil
3. Urban fear	Emotional cartography of urban fear: Methodological approach to analyse the relationship between built environment and fear of crime	Karla Barrantes-Chaves	2025	MethodsX	Multidisciplinary (methods and protocols)	Costa Rica
5. Redistricting	Creating Colorado’s 8th congressional district	Gabriella Subia	2024	Journal of Maps	Inter/multidisciplinary (maps and spatial diagrams across the physical and social sciences)	United States of America
6. Geo-Phenomenology	Geo-Phenomenology: A Qualitative and Humanistic GIS Approach to Exploring Lived Experience	Yuki Iwai	2024	The Professional Geographer	Geography	Japan
8. Emotional cartography	Emotional cartography as a window into children’s well-being: Visualizing the felt geographies of place	Andrew Steger, Elly Evans, Bryan Wee	2021	Emotion, Space and Society	Inter/multidisciplinary (theoretically informed research on the emotional intersections between people and places)	United States of America
14. Urban morphology	Urban morphological change in the case of Selcuk, Turkey: A mixed-methods approach	Seher Demet Kap Yücel & Gizem Aksümer	2019	European Planning Studies	Spatial or urban and regional development	United Kingdom
21. Neighborhood on tablet	A New Qualitative GIS Method for Investigating Neighborhood Characteristics Using a Tablet	Isabelle Schoepfer and Stephanie R. Rogers	2014	Carthographica The International Journal for Geographic Information and Geovisualization	Cartography, geovisualization, and GIScience	Canada

### 3.2 Interview selection and geo-indexical coding

Secondly, two members of the research team carefully reviewed the six in-depth interviews conducted during the original research conducted between October 2023 and January 2024. Following reflective discussions on the analysis focus with the full team, we concluded that two interviews were particularly relevant to the methodological goals of this paper. Specifically, these interviews provided rich material to address the challenge of operationalizing place-assemblages (Di Masso and Dixon, 2015), by identifying geo-indexical data that combined symbolic richness and spatial correlates.

For the analysis, we selected one interview with a community leader during the process of reclamation of the Quebrada, and another with a longtime resident of Peñalolén (see Table 1 for participant details). Both interviews contained a high density of geo-indexical references, allowing us to conduct a Reflexive Thematic Analysis (Braun and Clarke, 2019, 2021) aligned with the ontology of symbolic-material assemblages from the participants’ subjective perspectives. After familiarizing ourselves with the data (listening, reading, correcting transcription details and taking analytic notes), two independent coders identified every data fragment containing a geo-indexical reference (“here,” “there,” “the hill,” “Peñalolén”).

We define geo-indexicality by recalling the notion of ‘index’ - the property of a sign that gains meaning from the specific place and time in which it is situated. Similarly, ‘geosemiotics’ refers to “the study of the meaning systems by which language is located in the material world” (Scollon and Scollon, 2003: p. X). Based on these concepts, whereas recognizing that geo-indexical property acquires its full meaning when placed in the location to which it refers (which is facilitated by situated methodologies such as walking interviews, ethnography, etc.), we coded in each interview all narrative fragments that referred to ‘accounts opened up meanings tied to the spatial representation of a place’, considering both of them were produced in the district of Peñalolén, nearby the Quebrada. Each spatial reference was then interpreted to reveal its latent meaning.

We excluded only those references in which the spatial term referred exclusively to a temporal context (e.g., ‘there in the 70s’) or to places outside the area of analysis (outside Peñalolén and the hill).

The final corpus consisted of 143 coded data fragments (42 + 101), identified by two independent coders. These fragments were conceptualized as ‘units of meaning’- arbitrary but analytically grounded distinctions made by researchers during close reading of the material, aimed at identifying minimal yet meaningful segments for thematic development. As Braun and Clarke (2021) emphasize, a unit of meaning must “capture (at least) one observation, displaying (usually just) one facet” (p. 340).

Our analysis was conducted within a critical-interpretive paradigm, informed by principles of critical cartography and a psychosocial perspective on place-making processes. Coding focused on two interconnected levels:

- Spatial references: concrete locations mentioned in participants’ narratives, which would later be geolocated on the map; and
- Meaning-making processes- symbolic, affective, and discursive elements through which participants articulated their sense of place.

**MAP 1:**
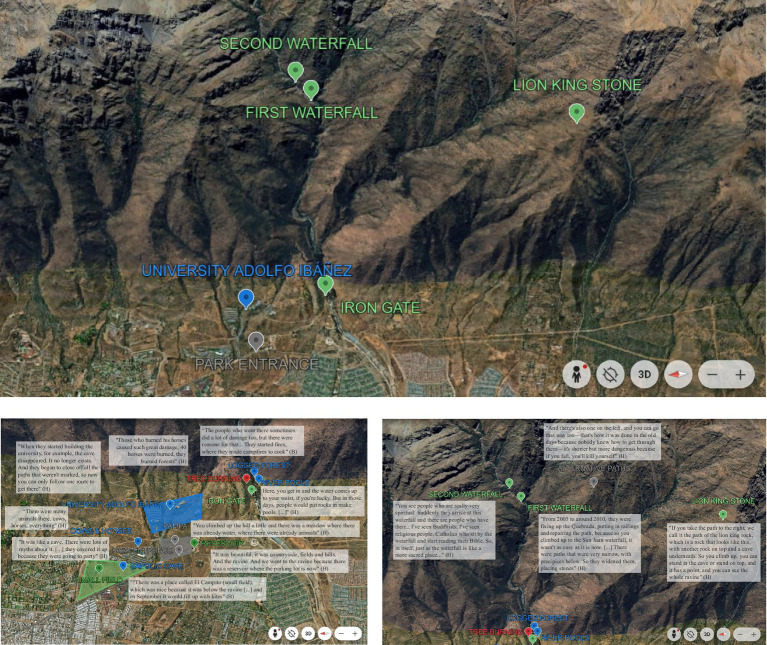
Historical and actual urban Peñalolén and hill. Green for places with positive emotional associations. Blue for places with nostalgic emotional associations. Red for places with an angry emotional association. Gray for places with a neutral emotional association.

All coded segments were subjected to one-to-one triangulation between coders to deepen and systematize interpretative rigor. The resulting themes were then collaboratively discussed with the full research team to ensure coherence and analytic depth. Table 2 presents the final coded units referring to the Quebrada de Macul Park exclusively, developed during this stage, which were subsequently used to construct the final cartographic representation of place-assemblages (see Map 1).

This analysis yielded two main interpretive themes: (I) The spatial frontiers between the park, the hill, and the city, appeared ambiguous in both interviews, not due to methodological limitations (e.g., poorly formulated questions or participants´ lack of spatial specificity), but because the ontology of place in this context is inherently characterized by the interweaving of the urban, the natural, and the inhabitants´ identities; (II) This overlap of social, subjective, and spatial layers is intersected with—and shaped by—the transformation of the territory over time.

This finding implies that only infinite temporal and layered cartographies could fully capture the complexity of the socio-spatial processes at play. Any map attempting to represent the psychosocial nature of this place must therefore introduce arbitrary boundaries and distinctions that simultaneously reveal and conceal power dynamics and lines of force. In this study, we chose to foreground the perspectives of the voices of those who have historically inhabited this territory- known in Chile as ‘*pobladores*’, a term referring to traditional residents of vulnerable neighborhoods who often organize collectively around community struggles.

In our sample, these voices are represented by a 79 years old woman (retired) and a 32-year-old man (worker), whose narratives articulate the lived memory and territorial identity embedded in the hill, the ravine, and the urban fabric of Peñalolén.

## 4 Results

### 4.1 Systematic literature review

A total of eight studies met the inclusion criteria and were analyzed in this review. Table 3 summarizes the main characteristics of these studies, including study ID, authors, title, year of publication, journal, and country of reference.

The selected studies were published between 2014 and 2025, with the majority appearing in the last 5 years (after 2020). They represent a range of geographic contexts - including Asia, South, Central and North America, and Europe - although they are more heavily concentrated in the global north. Most studies identified themselves as multi or interdisciplinary in nature, but the majority were published within cartography and geography journals, reflecting these disciplines’ growing interest in subjective and experiential dimensions.

Table 4 presents the methodological aspects of the studies included. Most adopted a qualitative methodological paradigm, typically grounded in constructivist epistemologies. Study designs varied, including case studies and mixed methods approaches, and sample sizes ranged from *N* = 1 to *N* = 128, with no consistent participant profile across studies.

**Table 4:** Methodological features in selected papers.

ID paper	Methodological paradigm	Design	*N* sample	Sample characteristics	Data collection	Data analysis	Quality	Use of technology for assemblage
1. Mapping migration	Mixed-methods approach	Enfasis on capturing “complexity” combining a Mixed-Method approach and Grounded-Theory	Does not disclose	Innmigrants detained in two detention centers, one in Mexico, other in Samos.	Participative maps, semistructured interviews, structured questionnaire.	Grounded Theory; does not disclose analysis for quantitative data, but all methods were triangulated.	Quality criteria for qualitative methods such as: (a) credibility, (b) transparency, (c) reflexivity.	Yes
2. Weaving nests	Qualitative approach	Qualitative, cartographic method (research-intervention) in a multiple case study (3). Considered inmersion in experience and existential territories.	*N* = 3	Users of a Psychosocial Care Center (CAPS) in São Paulo; adults over 40; low income (up to minimum wage); two men and one woman; vulnerable housing situations; long-term mental health treatment	Semi-structured interviews with affective maps (A2 sheets); city itineraries chosen and walked with researcher; field diaries; additional mapping using Google My Maps for trajectories (approximate geolocation for privacy)	Interpretive and reflexive analysis	They are not explicit, but there are: ethical criteria, reflexivity, triangulation.	No
3. Urban fear	Mixed methods.	Exploratory – Multiple case study	*N* = 128	Adolescents, adults, community leaders, community members, stratified by gender and poverty level, but not equally.	Walking interviews with groups of participants tracked with a GPS application and synchronized with the audio recording; semi-structured questionnaire; participatory mapping; focus groups; systematic observation (quantitative).	Thematic, discourse and spatial analysis	Yin’s four tests (construct validity, internal validity, external validity, reliability), piloting with a protocol, triangulation (in-depth interviews, observations), and a safety protocol for walks and field work (ethical issues on safety).	Yes
5. Redistricting	Qualitative methods	Single-case study design	2500 online comments. 150 map submissions. 350 h of hearings and meetings transcripts. 581 comments from participants in NDM. 45 walking/driving interviews.	Participants and commissioners in Colorado redistricting process; participants from NDM region (residence identified via ZIP codes); commissioners, legislative staff, and community members	Document analysis, content analysis, field interviews/observations. Compilation of online comments, map submissions, and transcripts (Atlas. TI coding); Ground-truthing via walking/driving interviews and site visits (coffee shops, parks, farms, community locations)	Manual coding in Atlas. TI; qualitative analysis of claims (method not disclosed); triangulation with ground-truthing	It demonstrates triangulation and traceability, but without a section on explicit quality criteria.	Does not attach subjectivity to the map
6. Geo-Phenomenology	Qualitative-dominant mixed methods	Qualitative case study using a mixed-methods approach (geo-phenomenology) combining phenomenology, NLP, and GIS. Single-case exploratory methodological case study	*N* = 1	University international student, lived in Japan for 3 years.	Two go-along interviews (day and night) on her commute route, recorded with GPS and audio. Transcripts were created from the recordings.	Morphological and sentiment analysis (NLP) of interview transcripts; Import of qualitative data (text, emotions, scores) into GIS for spatial analysis (kernel density, emotion mapping); Interpretative Phenomenological Analysis (IPA) of the spatial-emotional patterns.	Demonstrates procedural rigor and integration, not explicitly stated in the quality framework.	Yes
8. Emotional cartography	Qualitative descriptive exploratory	Conceptual/Self-Ethnographic. Authors declare that this is not an empirical study, rather, each of the authors leveraged their recollections of childhood to construct individual emotion maps.	*N* = 3	Authors of the paper	Each author produced a short narrative describing their emotion map, and specific quotes emphasize how emotional attachments are produced in their interactions with the world. Specifically, a ‘draw-and-write’ approach. They focus on the embodiment of feelings in the emotion maps they create.	Authors use self-constructed emotion maps to recall and interpret the felt geography of our childhood places	Theoretical coherence, reflexivity, and transparency	No
14. Urban morphology	Mixed-methods approach	Single case study	*N* = 15	9 men and 6 of women, had lived in the city for at least 25–30 years.	Documents and cartographies analyses (historical maps, aerial photographs, Google Earth image). Qualitative: commented walks (mobile ethnography with phenomenological sensitivity) to elicit perceptions, memories, and meanings of change.	Cartographic analysis (quantitative–spatial). Conzenian historical-geographical approach: identification of morphological periods through GIS redrawing; intertemporal comparison to see city limits, growth, transformed natural resources. Output: vector layers by date/period describing the formal change in the fabric. Qualitative analysis (narratives and memory) Thematic coding of stories (according to Neuman): organization of themes, places, and dates first in Excel (frequencies and basic taxonomies). Key result: layer of “Lost Selçuk” (disappeared places) and files per site with before/after, period, number of mentions, etc.	Triangulation. No explicit criteria	
21. Neighborhood on tablet	Qualitative	Single case study (Geneva) of an exploratory-descriptive nature.	*N* = 16	Young professionals (aged 25–40) with higher education and living in Geneva, Switzerland.	Semi-structured interviews accompanied by a digital map: customized maps are produced during each interview (drawings of points/lines/polygons on iPad/ArcGIS) along with verbal explanations given while drawing (audio recorded) and written comments on the attributes of each object.	It does not report formal thematic coding with qualitative software or statistical metrics; its analysis focuses on spatial and comparative readings of the drawings, supported by verbal explanations and attributes collected during the interview.	Reflexivity	Yes (tablets and gis)

In terms of data collection, walking interviews supported by tracking devices to record the participants’ trajectories were the most frequently employed techniques. Data analysis procedures were primarily interpretative or not explicitly described. The use of digital technology was reported in 5/8 of the studies, most often related to GIS applications, although more participatory techniques – such as hand-drawn maps or the placement of stickers on a map- were also used at certain stages.

Table 5 synthesizes the main findings, authors’ discussions, and thematic interpretations across the reviewed studies. Results highlighted a broad range of outcomes, illustrating how these methods can shed light on diverse phenomena. Overall, the studies demonstrate that emotions are deeply embedded in place: looking at the lake soothes feelings of loneliness; certain environmental elements can heighten fear depending on the social group; and children embody and cultivate emotional bonds with their surroundings. Discussions were consistent with these empirical results, highlighting the persistent challenges inherent in translating subjective experiences into cartographic representations.

**Table 5:** Analysis of results and discussions held in selected papers.

ID paper	Principal results	Discussion in the paper	Interpreted topics
1. Mapping migration	Emotions in detention centers were mapped using colors and symbols, revealing unsafe areas linked to institutional practices.	Mixed-methods design faced contradictions between qualitative and quantitative data, though color mapping revealed dominant emotions.	Data assemblage was partial but showed how institutional elements (e.g., cameras, guards) shape emotional geographies.
2. Weaving nests	Maps included participant-drawn trajectories and geographic references, capturing subjective perceptions with little interpretation.	Affective mapping engaged participants as co-researchers and highlighted exclusionary dynamics in urban space.	Despite minimal interpretation, participant-generated maps offered nuanced subjective insights.
3. Urban fear	Maps showed spatial distribution of fear, linking it to gated areas and gender differences.	The method enabled comparisons across gender and time, especially following environmental changes.	Sampling was rigorous; maps integrated multiple perspectives but offered limited emotional nuance.
5. Redistricting	Maps tracked redistricting and industrial locations, revealing links to community interests.	Historical policies shaped geographic change and residents’ sense of place, but methods lacked insight into daily interactions.	Focused on legal and spatial boundaries, with qualitative meaning added through text rather than cartography.
6. Geo-Phenomenology	Maps represented sense of place and emotional density, revealing how place supports migrants coping with loneliness.	GIS tools enriched geo-phenomenological analysis and highlighted belonging and attachment.	Maps prioritized subjective meaning over spatial accuracy, projecting assemblages effectively and intuitively.
8. Emotional cartography	Mapped “felt geographies” showed how children form emotional bonds with their surroundings.	Traditional cartography marginalizes subjectivity; emotional cartography centers children’s perspectives and promotes empathy.	Including subjective experience reveals deeper place attachments — a core of place-assemblage.
14. Urban morphology	Commented walks revealed fine-scale urban changes not visible in quantitative data.	Understanding morphology requires memories and lived experiences, not just physical data.	Combining historical maps with narratives shows how spatial change is also symbolic and experiential.
21. Neighborhood on tablet	Tablet-based GIS enabled participants to create spatial data and reflect on “neighborhood.”	Interactive mapping acted as a cognitive and dialogical catalyst, requiring renegotiation of boundaries.	Technology provided direct access to subjective spatial perceptions through participant-generated content.

**Figure 2:**
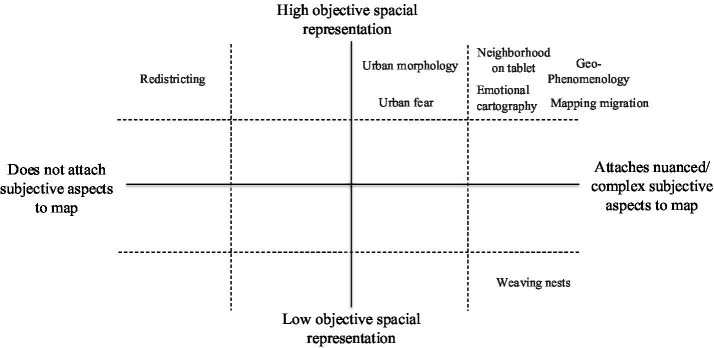
Cartesian map to classify selected papers.

In conclusion, the methodologies employed across studies varied in terms of the levels of objectivity represented on maps – for example, in precisely real distances or environmental features are depicted - and the degree of nuance achieved in capturing subjective experience. These ranged from minimal integration of subjectivity into maps to richly textured representations of lived experience (see Figure 2).

The interpreted topics clustered around several key dimensions (Figure 2 [cartesian plan]). Most studies prioritized spatial objectivity while managing to incorporate some degree of nuanced subjectivity, demonstrating how different methodologies can successfully achieve this balance. Only one paper explored subjectivity without spatial objectivity by relying exclusively on drawings (although it did present an objective map of the locations mentioned by participants, this lacked any subjective layer). Another study failed to map subjective dimensions but used advanced software to objectively spatialize participants’ interests (e.g., oil and gas industries), which aligned with the interpretive discussion in the text. Overall, literature appears to converge on a shared emphasis: the use of objective maps to represent nuanced subjectivity- though the means to achieve this objective vary considerably. As we can see in the figure, work may have low use of technology but a high level of subjective complexity (such as the work of Silva, 2022), or heavy use of technology and a low level of subjectivity projected on the map, such as the work of Subia (2024).

While the systematic review highlights diverse methodological strategies for representing subjectivity in cartographic form, these studies also reveal persistent gaps and tensions. Specifically, they underscore how challenging it is to integrate the affective, symbolic, and lived dimensions of place into spatial representations without imposing rigid cartographic boundaries. To explore these methodological challenges in greater depth, we turn to our own empirical material: two in-depth interviews with long-time residents of Peñalolén. This analysis examines geo-indexical narratives as an entry point for mapping place-assemblages, allowing us to observe how spatiality is embedded in personal histories, collective identities, and everyday experiences.

### 4.2 Methodological challenges of place-assemblages in people’s experiences: analyses of geo-indexicality within two in-depth interviews

As mentioned earlier, our thematic analysis revealed two overarching interpretive dimensions that cut across the interviews: (1) the ontological indistinction between park, cerro’ (hill), quebrada’ (ravine), and city, and (2) the temporal layering of social, spatial, and subjective dimensions that shape place identity over time. In what follows, we present each of these themes, illustrating them with participants’ narratives and exploring their psychosocial significance.

#### 4.2.1 The ontology of a place shaped by people’s experiences

It was noteworthy that the first distinction to emerge among the codes was temporal rather than spatial: fragments referred either to the past or the present state of place. Although one interview was conducted with an older person (79 years old) and the other with a younger participant (32 years old), approximately half of the geo-indexical elements described the past, depicting the cerro (hill)—without clear distinctions between quebrada (ravine) or park—as a continuous space with the municipality of Peñalolén and its urbanization process.

“This was all cerro. Horses roamed free, they were covered with quilo, quilo was like a vine, many thorn bushes. And the only houses there were in Río Claro. And José Arrieta was paved, but only on one side — the side that belonged to La Reina.” (Bernardita, p. 6, own translation)

“[…] To begin with, Grecia Street didn’t exist. Upwards there was nothing, and later on… there were informal settlements, then the Grecia road was built, and they reached where the Quebrada was, but even then, it was still cerro.” (Hernán, p. 24, own translation)

In both interviews, participants spontaneously began describing the quebrada in historical terms and biographical terms, narrating how they personally witnessed the area’s transformations over time. Bernardita, as a political leader, situates the ravine’s transformation within the urbanization of Peñalolén Alto, while Hernán recalls childhood summers spent on the ‘cerro’. This shared perception of spatial continuity reflects how lived experience precedes geopolitical distinction.

“… and we used to come here, and the bus dropped us off at Plaza Egaña. And from there we came with everything, with tents. We came camping during the summer. Up there was the school, where SEK University later was. It used to be a school. So, as I said, we came here to spend the summer — it was our vacation spot for about a month.” (Bernardita, p. 6, own translation)

“When we went, I don’t know, to the ‘Sapolio Cave’, it’s next to your university. […] It was like a cave. There were a lot of myths, from what I heard: ‘No, it’s a caveman’s cave’, ‘there was sapolio there in the cave’. I went, I knew it, and yes, it was a cave. Even today, sometimes we go up and pass by your university, and it’s still there, but it’s covered. [How covered?] Covered with earth. They covered it, but you can still see a bit of it… the cave was there, and yes, they covered it because people used to go there to party.” (Hernán, p. 24–30, own translation)

In fact, the distinction between cerro and city seemed almost nonexistent. Bernardita recalling her early years in the upper part of Peñalolén during the urbanization process:

It was hard. We had no water. A water truck would come later to deliver water into barrels. […] We drew water from the Las Perdices canal because the water was our swimming pool, the Las Perdices canal. The water was transparent, clean — not for drinking, but for washing. And since I loved plants, I’d dig here, ohhh, and fix the soil — it was all clay here, this was Peñalolén. [Why, how so?] It was all clay, hard clay. It would dry and harden. It would get hard. So, we fixed it with leftover tea — we’d even make the tea in small teapots. Anything that could improve it… sand when we could get some. And later we started bringing litre soil from up there. But we had to sneak it in. (Bernardita, p. 30–34, own translation)

This indistinction between cerro and city is rooted in a period when urbanization had not yet fragmented the territory. The cerro functioned as a shared, undifferentiated space for neighbors and summer visitors- a background used for leisure and everyday activities. This supports the idea of urbanization “consuming” the cerro—dissolving countryside and city boundaries.

The lack of distinction among cerro, quebrada, and park points to a deeper ontological fact: the place we know today as Quebrada de Macul Park was born from social practices and meanings attributed to it by neighbors. It emerges in discourse before it does in geopolitical maps, reflecting a relational rather than a bounded spatial understanding.

We also go up there and it’s like, ‘Hey man, if you’re going to smoke a cigarette, don’t throw it into the water, you know?’ ‘Ah, yeah, sorry.’ We really take care of our cerro, and we have friends too who go up there and pick up trash. I mean, we take care of our cerro because we live at the foot of it, you know? (Hernán, p. 259, own translation)

“V: That’s really interesting, Hernán, what you said — that you and your group of friends see it as ‘our quebrada,’ like it belongs to you.

H: Yes, it’s ours.

V: Is that something you feel within your group of friends, or have you heard it from other groups in Peñalolén?

H: No, that’s just how Peñalolén is. In the end, for example, if you go to Violeta Parra or to the other houses near the cerro, they really take care of their cerro. The ecological community, Camino La Rosa.

V: And they all have that sense that it’s theirs.

H: Of course, it’s their cerro. Our cerro.” (Hernán, p. 297–302, own translation)

For the inhabitants of Peñalolén, it has been “their” cerro since the municipality’s origins. The distinctions between the cerro, the city at its base, and what later became the Quebrada de Macul Park are subsequent constructs introduced mainly by external actors, including the research team.

“T: Hey, when you say, Hernán, ‘what the community values most,’ what do you mean by the community?

H: Peñalolén, the Quebrada, the cerro.” (Hernán, p. 256–257, own translation)

**Figure 3:**
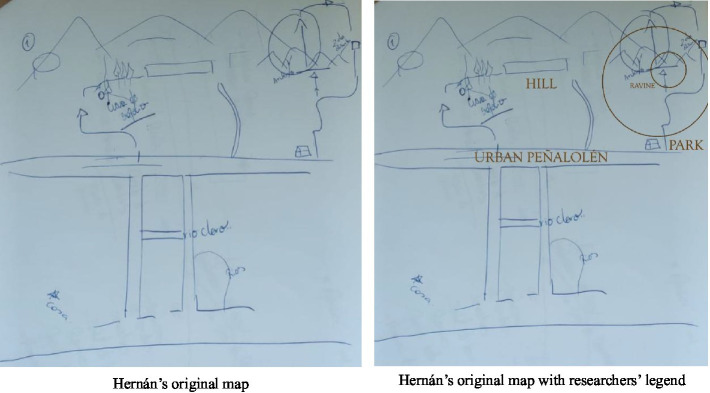
Participant’s spontaneous mental map **(a)**. Participant’s spontaneous mental map with legend **(b)**.

Our Reflexive Thematic Analysis shows that the Quebrada de Macul Park is built upon articulations of indistinct places—if we understand “place” as a socially and subjectively imbued space—defined more by the identity than by geomorphology. In this sense, the distinctions made by the research (cerro, quebrada, and park) are analytical rather than experiential. From the neighbors´ perspective, everything is “their cerro.” These (in)distinctions appear in the mental map produced by Hernán, as he remembered and narrated the places:

As Figure 3 illustrates, geopolitical distinctions between park, ravine, hill and city appear only when applied externally by researchers. Identity and place are intertwined as a whole—a form of mimetic place identity, where speaking about place is subjectively equivalent to speaking about the self. As Ropert (2019) notes, “being there implies being.” Moreover, these distinctions are historically produced and evoke ambivalent affects such as nostalgia and anticipation. The absence of distinctions can also be interpreted as a form of resistance to transformation. Time–space distinctions in place identity: when the place arises between nostalgia and collective action.

As highlighted in the previous section, the two interviews analyzed reveal a deep entanglement between place identity and place itself. Distinctions in-place are constructed through perceptions, emotions, and memories out-of-place as participants narrate the story of Parque Quebrada de Macul from their homes. Everything seems layered and overlapping. Bernardita’s place identity is inseparable from her identity as a political leader who fought for housing during the urbanization process; Hernán’s identity is tied to his role as a historical neighbor who hosts and guides others in the park. The recurring notion of “our cerro” reflects an identity – centered abstraction that foregrounds belonging over transformation.

**MAP 2:**
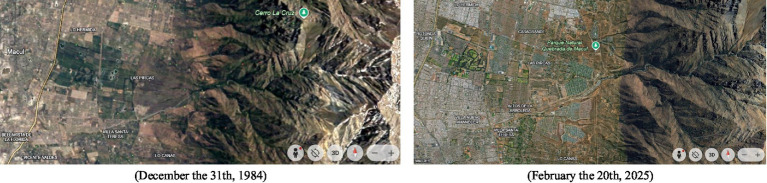
Place-assemblages’ map.

From the perspective of the urbanization process described by our interviewees (see Map 2 above for a reference of landscape transformations due to urbanization processes)—which profoundly transformed life at the foot of the cerro while still preserving a sense of continuity between the natural and built environments (see previous section)—spatial distinctions increasingly referred to urban elements rather than the cerro itself. Participants named places such as José Arrieta Avenue, SEK University, the regiment, the first healthcare center, and the first supermarket as key spatial landmarks.

We had a small healthcare center, just a house in front of the regiment. That was our healthcare center. Later, well, it grew. … And then we moved there. […] And then we started to organize ourselves with the neighbors. In front, there was nobody, nobody, nobody. We were alone. And the fences were just barbed wire. They were only used to mark the lot sizes. And there was one or another small grocery store, that’s where we got our supplies […]. […] Until later, over time, they put up a Unimarc. Unicop, Unicop, Unimarc, I think it was called. It was on Río Claro. (Bernardita, p. 16–24, own translation)

Some spatial distinctions associated with the cerro and quebrada also began to appear during this period, including the reservoir (‘*tranque*’), the ‘*campito*’ (small field), the Sapolio Cave, the area of ‘*litre*’ trees and old trees (which people sometimes burned out of malice), the pampa, and the alternative paths to go up the mountain (see Map 1).

A particularly revealing finding is that urbanization coincided with the emergence of spatial demarcation—a process shaped by the informal occupation of privately owned land and the influx of new residents. Access was not directly restricted but regulated through a planning logic that inherently defined who could and who could not enter certain areas.

Later on, over time, they started putting up wire, closing it off, they wouldn’t let us through, there was no way through. (Bernardita, p. 113, own translation)

[And was that the way you used to go up to the Quebrada?] Exactly, there were no fences, nothing, and of course, from there it was different because you reached the cave, continued diagonally past where your university is, and all of that was like a plain. And there was a different kind of path there — now that path is closed. There’s a guard, it’s fenced off, you know? (Hernán, p. 38–40, own translation)

Participants also alluded—albeit subtly—to the diversion of water sources, again reflecting spatial transformations linked to urbanization and the enclosure of the area for private use, whereas concerning environmental justice issues:

We spent like two hours putting in big rocks and all that to make the dam so we could bathe — the rocks were huge, and now they look even bigger because before you could only see the parts that stuck out of the water, but now, no, now there’s so little water that it’s sad … but that water was diverted when they started buying the plots up there … [Ecological Community] They screwed us with the diversion… they built a canal, a little canal, and diverted the water. (Bernardita: p. 121-125, own translation)

Finally, with the advance of urbanization, the cerro acquired new spatial distinctions associated with the construction of the park, which profoundly transformed how neighbors interacted with place, generating ambivalence in their subjective experiences. The construction of designated trails, a guardhouse to control access, and the introduction of a buggy to transport injured visitors all improved safety while simultaneously regulating and restricting uses of the natural space.

Well, from 2005 to around 2010, they also improved the Quebrada in the sense that they started installing railings and fixing the path because, as you went up to the San Juan waterfall, it wasn’t as easy as it is now. […] So there were paths that were very narrow, and below there were cliffs. So they widened them, placed stones. (Hernán: p. 88–90, own translation)

The role of the university—where most members of the research teamwork or study—was described as controversial by participants. On the one hand, the institution played an active role in urban transformation; on the other, it was perceived as disengaged from the cerro’s development until it became a metropolitan park.

It kind of annoyed us because, well, ‘Why did they block this passage?’ So we said, ‘Maybe because of the animals, the breeding grounds, or maybe the university bought this space and wants it for themselves to keep building things,’ because we haven’t gone all the way up — I’ve never been inside that university. I think just once, but I didn’t go that far. (Hernán: p. 141, own translation)

“Yeah, I’ve noticed that, for example, at the foot of your university, it’s super dirty, you know? Like sometimes when I’m coming down, I see students going up, and they say, ‘Wow, yeah, the cerro is beautiful, but why is there all this garbage here?’ And there’s a lot, tons of garbage. […] So… I also wonder, could the university manage a debris truck? And take all that away. Maybe they could have a truck once a month or every so often — after all, people are dirty. […] They have the purchasing power to do it. I mean, they bought the entire cerro.” (Hernán: p. 278–282, own translation)

In summary, the new places that emerged with the construction of the park—such as trails, the San Juan waterfall, guardhouses, signage, the university, additional waterfall, hazardous areas, and alternative paths (see Map 1)—illustrate a process of spatial-use diversification that simultaneously acts as a mechanism of bordering, demarcation, and definition. This process inevitably transforms what once mimetic place identity –defined as when “being there implies being” (Ropert, 2019), into a more fragmented experience, progressively distancing people from the cerro.

## 5 Conclusion and discussion

This study examined methods for rendering subjective experience cartographically, asking: (How) Can subjective and/or psychosocial processes embedded in people-place relations, be cartographically represented? Our answer, grounded in a systematic review and a reflexive thematic analysis of two in-depth interviews, is threefold. First, subjectivity can be mapped, but doing so entails inevitable trade-offs between spatial objectivity and the nuance of lived experience. Second, representations are strongest when they are ontologically place-assemblages, integrating affective, narrative, embodied, and material registers. Third, every narrative contains space: stories are never placeless; they are anchored in-and help constitute the spatial imaginaries through which experience, emotion, and meaning are organized.

### 5.1 Systematic review

We found that the studies identified are relatively recent (since 2014), with a noticeable concentration around 2020, paralleling the growth of online geo-indexing and story-mapping tools since 2010 (Caquard and Dimitrovas, 2017). This aligns with an epistemic shift in human geography, urbanism, and the social sciences, which increasingly places subjectivity and sociopolitical processes at the center of how space and the city are represented and conceptualized. Traditional views of landscape as a naturalized and stable social representation of the physical environment have been challenged by accounts that emphasize processuality, embodiment, and cultural variability (Di Giminiani and Fonck, 2015). As Massey (2008) suggests, “perhaps places do not lend themselves to having lines drawn around them” (p. 22): places should no longer be conceived as clearly bounded spaces but rather understood as relational nodes, within the framework of geographies of dependencies, where the local and the global are mutually constituted through interconnections and power relations.

This shift is consonant with a post-representational cartography, which privileges map-making as more significant than the final product, with meaning emerging from its use in specific contexts and for particular purposes (Caquard and Cartridge, 2014). Visual storytelling variously reasserts the situated, subjective, and intentional character of cartographic design, a trend evident in the journals where these studies were published- even though many of these journals originated in fields such as geography or cartography, they increasingly feature work focused on subjective mapping.

Yet, despite improved computational tools, mapping subjectivity remains challenging. We observed recurring gaps: the number of people represented on a single map (Barrantes-Chaves, 2025); the depiction of sociodemographic characteristics (Barrantes-Chaves, 2025); technology choices and their implications (see Silva, 2022, versus Iwai, 2024); temporality (past, present, and future; Subia, 2024); and the quality of qualitative-quantitative integration in mixed methods (Manek et al., 2023). Caquard and Dimitrovas (2017), further highlight that deciding what to project- and what to omit- renders the researcher’s role methodologically central.

Plotting the reviewed studies on our Cartesian plane—(1) level of spatial objectivity and (2) level of projected subjective complexity—reveals that, while some still struggle with the classic dichotomy between objective representation and subjective complexity (e.g., exhibiting high objectivity and low subjectivity, or vice versa), most succeed to integrate both dimensions to varying degrees. Although the concept of place-assemblage is not explicitly invoked, the overall trend suggests that current research is advancing in this direction from interdisciplinary perspectives (including Psychology, Public Health, Geography, Urban Development, and Methodology).

It is also evident that most studies cluster in the quadrant representing high subjective complexity and high objectivity, while those with high subjective complexity and low spatial objectivity are relatively scarce. This suggests a persistent preference for objective representations of space over more phenomenological approaches. For instance, Silva’s (2022) work—which presents participants’ hand-drawn routes between the mental health institution they attend and their homes—offers a rich and complex interpretive framework. Yet, most studies rely on maps generated through software. When this choice is not justified by the project’s objectives, it may reflect an epistemological or ontological bias within the discipline toward quantitative precision, or perhaps a methodological limitation of the researcher’s field—indeed, psychology was the only field to apply hand-drawn mapping.

Beyond these factors, the challenges described above also point to additional axes intersecting the Cartesian plane, which further complexify the cartographic representation of subjectivity. A study may vary in its capacity to project emotions, represent population size, capture temporal dimensions, or group differences, among others—together forming multidimensional representational spaces.

### 5.2 Reflexive thematic analysis of interviews

Our empirical analysis highlights an insight that both constrains and enables cartographic work: all textual accounts are spatial because every narrative contains space. People’s accounts are never placeless; rather, they are anchored in and shaped by specific spatial references, whether these are explicit or implicit. This spatial embeddedness is not merely descriptive, but constitutive of how meanings are produced, identities formed, and experiences remembered. In this sense, each interview functions as a spatial text, where stories and places are co-constructed. However, it is also important to acknowledge that this is true of the other side of the relationship: as Lefebvre (1991) notes, the concept of ‘counter-space’ is key to highlighting the two-way relationship between place and historic social relations. Therefore, we must remember that places are products of unequal forms of power, social relations, and conflicts, and maps should be able to condense these concrete, situated properties, as opposed to abstract, homogenizing ones.

Moreover, Results had examined how these geo-indexical anchors reveal the lived ontology of Parque Quebrada de Macul, highlighting how spatial distinctions—or their absence—shape people’s place attachments and experiences over time. The interviews revealed that distinctions that appear self-evident to external observers - such as those between the hill, the park, and the ravine, or between the municipality of Peñalolén and the hill- were not primary distinctions for participants. Instead, boundaries emerged historically through processes of urbanization. Abstract references to place—such as speaking of the “hill” without clearly defined spatial boundaries—illustrate how the continuity of lived experience can blur geopolitical demarcations, rendering cartographic representation more diffuse. This difficulty resonates with the challenge of adopting a “top-down” cartographic perspective while being *in situ* (Ingold, 2011; Iturra Muñoz, 2014). The Renaissance bourgeois concept of landscape as something external, contemplated from afar, strains the possibility of understanding landscape as incorporating human experience- an experience produced through co-creation, imagination and creative action (Imilan and Iturra, 2014). More recent perspectives conceptualize landscape as a process of life itself, allowing classic dichotomies to be overcome and revealing it as a collective manifestation of ideological values specific to particular geohistorical contexts (Di Giminiani and Fonck, 2015).

From this standpoint, our attempt to map place-assemblages is necessarily fraught with tension. The more complex the object- intertwining social, material, and discursive elements (Di Masso and Dixon, 2015)- the more difficult it becomes to stabilize a fixed representation. Capturing such complexity would require mapping potencially infinite layers. This points to a fundamental ontological challenge: if place is understood not a set of fixed containers but as an assemblage -an entanglement of social, material and discursive processes the very possibility of definitive cartographic representation is destabilized. For instance, studies of everyday mobility and activity spaces show that people’s perceptions of diversity do not necessarily mirror demographic or “statistical” indicators. Instead, perceived diversity is shaped by lived patterns of intergroup contact, affective affinities with places, and broader attitudes toward outgroup members, highlighting the experiential and relational nature of how diversity is interpreted in urban spaces (Holubowska et al., 2025).

Similarly, recent studies show that psycho-affective dimensions impact the nature of people-place relationships, showing how spatial narratives, symbolic elements, and built form jointly contribute to emotional experience and identity formation within specific socio-historical contexts (Shi and Tian, 2025). In the same vein, affective mapping studies demonstrate how processes of appropriation and expropriation mobilize emotions such as belonging, agreeableness, and contrast, revealing the depth and ambivalence of person–place ties in situations of urban threat or displacement (Pinheiro Pacheco and Cruz Bomfim, 2025). Urban research further indicates that such emotional and symbolic dynamics unfold within historically layered spaces, where past inscriptions and contemporary practices interact conflictual ways. For instance, studies on urban resignification reveal how transformations in the built environment activate tensions between heritage, everyday uses, and emerging imaginaries—producing contested meanings and renewed affective attachments (Jiménez-Pacheco et al., 2025).

However, our results call for a deeper discussion of the onto-epistemological issues involved in cartographies of place-assemblages. First, it is important to acknowledge that we are working with a multifaceted object of considerable complexity, one that requires a multilevel understanding of tightly intertwined components, This situates our methodological proposal within the post-constructionist turn, which rejects not only the representationalism of classical psychology but also advances the view that psychosocial objects emerge from social practices and are traversed by historical and political dimensions that often lie at the root of inequality (e.g., Gergen, 1998; Iñiguez Rueda, 2003; Pavez Correa, 2021; Piper Sharif, 2008).

Within this frame, geographies of encounter sharpen the methodological stakes. Public space is a site where embodied social distinctions, identities, and forms of belonging are continuously negotiated. Encounters in urban contexts—far from being trivial or fleeting—can foster social cohesion, generate new forms of recognition, and legitimize plural identities (Aelbrecht and Stevens, 2018). Cartographies informed by the concept of place-assemblages enable psychology to capture narratives and embodiment simultaneously, moving beyond text-only accounts to show how bodies, artifacts, and boundaries organize everyday life. Methodologically, this effort is worthwile insofar as it helps consolidate the onto-epistemological nature of place-assemblages, while practically, it may also equip publics and decision-makers, with visual tools for action in the spirit of critical and counter-cartography.

Thirdly, the onto-epistemological challenge addressed in this paper responds to a growing dissatisfaction with the limited practical impact of environmental psychology, a field still largely dominated by mainstream quantitative methodologies (Kuhn and Bobeth, 2022). Calls for environmental psychology to engage more explicitly with ideologies and power structures (Kuhn and Bobeth, 2022) resonate with critical perspectives that emphasize the need to recover an understanding of the subject as situated within historical and political contexts (Di Masso, 2012; de Pinto Carvalho et al., 2025). This stance is crucial for the type of research presented here, in which cartographic representation must serve to situate experience within a multilevel context rather than abstract it from its conditions of emergence. The challenge, therefore, lies in constructing maps that do not simplify complexity, but instead articulate it. In this sense, maps are conceived as action-oriented tools capable of revealing the intertwined processes through which collective action unfolds. To avoid remaining confined to momentary or decontextualized analysis, Kuhn and Bobeth (2022) suggest working with biographical approaches – an orientation that aligns closely with our proposal to cartographically represent situated individual historical narratives. One open question that remains concerns the sample size capable of sustaining this level of complexity, an issue that poses a challenge for future research initiatives.

Taken together, these considerations lead us to conclude that representing assemblages is “conceptually flawed,” if the aim is understood as producing static representations. Rather, the task is to develop not to “represent” but to produce new cartographic approaches to place-assemblages. Our interviews suggest that abstraction becomes particularly problematic when: (i) speaking from the outside (without lived involvement) or (ii) speaking individually (without collective negotiation). By contrast, it is through collective processes that distinctions materialize, places become embodied, and references are named. The place-assemblages of the hill—today known as Parque Quebrada de Macul—are precisely anchored in participants’ lived experiences, yet they become increasingly artificial when abstracted from the narratives of those who have linhabited and transformed this landscape over time.

Subjective and psychosocial processes can be addressed most effectively when researchers (a) treat maps as processes rather than final products; (b) begin from place-assemblages, integrating narrative, affective, embodied, and material traces; (c) embrace mixed methods (e.g., walking interviews, hand drawn maps, GIS layers, and geo-indexical coding); and (d) make methodological trade-offs explicit, recognizing that gains in spatial precision may come at the cost experiential texture, and vice versa. In short, researchers should first identify the relationships they seek to reveal, and then select the least distorting methods to address those relationships, regardless of its elegance. This pragmatic orientation, aimed at recovering voices from within the territory itself, is what ultimately guides this methodological exercise and gives it its true value.

### 5.3 Limitations and future directions

Throughout this process, we repeatedly confronted the temptation to misrepresent place-assemblages by reducing them to one or two of their constitutive dimensions. Similarly, synthesizing the reviewed studies required organizing their contributions along two analytical axes or coordinates, an operation that inevitably simplified complex methodological and theoretical proposals. Building on the critical cartography approaches discussed above, we argue that these debates risk becoming sterile unless grounded in a pragmatic purpose. In this study, that purpose is the recovery of living memory in a historically transformed territory, making visible power asymmetries and competing discourses. This stance aligns with participatory mapping traditions in digital platforms (e.g., carto.com, openstreetmap.org, mapkibera.org) which foreground plural voices within cartographic representations (Kitchin and Dodge, 2007; Wood and Fels, 2008; Caquard, 2013), as well as with participatory action research, showing the methodological and political potential of collaborative mapping practices (Kindon et al., 2007).

Nevertheless, our findings are constrained by several limitations: (i) the systematic review excluded gray literature in order to focus on mainstream academic debates, despite the fact that many significant contributions – particularly in critical and participatory cartography – emerge from local and practice-based contexts, (ii) the analytical research team was originally made up exclusively of psychologists; although an urbanist contributed to the theoretical discussion and conclusions, the study would clearly benefit from deeper interdisciplinary discussion. These limitations underscore the need for collaborative, multimodal and interdisciplinary approaches capable of developing representations of place-assemblages that more fully capture subjectivity-in-place (including temporality, scale, multiplicity of voices, and power relations). Future work should systematize geo-indexical coding protocols, expand participatory mapping practices that return cartographic outputs to communities, and test hybrid pipelines (hand-drawn → digitized → layered interpretations) that preserve experiential nuance while achieving spatial legibility.

In closing, we return to a central insight emerging from our results: every narrative is spatial. The imaginaries we analyze are always situated and inseparable from experience, emotion, and meaning. When cartography, is practiced from an ontology of place-assemblages and approached as a participatory and reflexive craft, it can render these imaginaries thinkable and actionable—not by fixing them in space, but by opening them to collective presentation, interpretation and transformation.

## Data Availability

The raw data supporting the conclusions of this article will be made available by the authors, without undue reservation.
