# Correction: Mapping meanings: methodological innovations to restore memories of place in a public park in Santiago de Chile

**DOI:** 10.3389/fpsyg.2026.1833696

**Published:** 2026-04-07

**Authors:** Teresa Ropert, Ana Rosenbluth, Martín Nilo, Luis Valenzuela, Michelle Bernardino

**Affiliations:** 1 School of Psychology, Escuela de Psicología, Universidad Adolfo Ibáñez, Santiago, Chile; 2 Escuela de Gobierno, Universidad Adolfo Ibáñez, Santiago, Chile; 3 Independent Researcher, Río de Janeiro, Brazil

**Keywords:** critical cartography, environmental psychology, geo-indexicality, place-assemblage, qualitative methods, visual methods

The order of authors in the author list of the published paper was erroneously given as: Luis Valenzuela, Ana Rosenbluth, Martín Nilo, Teresa Ropert and Michelle Bernardino.

The correct author list reads:

Teresa Ropert, Ana Rosenbluth, Martín Nilo, Luis Valenzuela, Michelle Bernardino.

In the published article, ORCID information was missing for author Teresa Ropert, Martín Nilo, Luis Valenzuela, Michelle Bernardino.

This has now been added for author Teresa Ropert (https://orcid.org/0000-0001-5020-1191), Ana Rosenbluth (https://orcid.org/0009-0001-5482-0747), Martin Nilo (https://orcid.org/0009-0000-1584-0035), Luis Valenzuela (https://orcid.org/0009-0008-5672-7563), and Michelle Bernardino (https://orcid.org/0000-0001-6494-755X).

The original version of this article has been updated.

